# A Sulfated Polysaccharide from Red Algae (*Gelidium crinale*) to Suppress Cells Metastasis and MMP-9 Expression of HT1080 Cells

**DOI:** 10.3390/foods11152360

**Published:** 2022-08-06

**Authors:** Haiyan Zheng, Yu Pei, Yuan-Lin He, Yi Liu, Minqi Chen, Pengzhi Hong, Chunxia Zhou, Zhong-Ji Qian

**Affiliations:** 1 Guangdong Provincial Key Laboratory of Aquatic Product Processing and Safety, Guangdong Province Engineering Laboratory for Marine Biological Products, School of Chemistry and Environment, College of Food Science and Technology, Guangdong Provincial Engineering Technology Research Center of Seafood, Guangdong Ocean University, Zhanjiang 524088, China; 2 Southern Marine Science and Engineering Guangdong Laboratory, Zhanjiang 524025, China

**Keywords:** sulfated polysaccharides, *Gelidium crinale*, MMP-9, migration, invasion

## Abstract

Sulfated polysaccharides from red algae have a variety of biological activities, especially antitumor activities. Matrix metalloproteinase-9 (MMP-9) is a proteolytic metalloenzyme that degrades the central part of the extracellular matrix (ECM) and promotes tumor metastasis. In this research, we have investigated the influence and mechanism of GNP (sulfated polysaccharide from *Gelidium crinale*) on tumor metastasis and MMP-9 expression of human fibrosarcoma (HT1080) cells. The results inflected that the concentration of GNP below 100 μg/mL has no toxicity to HT1080 cells, but showed excellent activity in inhibiting cells migration and invasion. In addition, GNP effectively inhibits the mRNA of MMP-9 and reduces its expression and activity by regulating nuclear factor-kappa B (NF-κB), mitogen-activated protein kinases (MAPK) and mTOR/PI3K/Akt signaling pathways. GNP has great potential as MMP-9 inhibitor and could be developed as a functional food or drug to prevent tumor metastasis.

## 1. Introduction

Tumor is a mass caused by abnormal cell division, which seriously threatens human life and health [1]. It is usually defined as two types: benign or malignant, and cells in malignant tumors may spread to surrounding tissues or other parts of the body, thereby leading to metastatic cancers [2]. Tumor metastasis is the main obstacle to the success of tumor prevention and treatment, and accounts for 90% of tumor-related deaths [3]. Tumor cells detach from the primary site, digest the ECM, and migration of the cell takes place, then intravasation into blood or lymph vessels and transport throughout the organism, finally metastasize to a distant site and form the same type of tumor [4]. Metastasis is a multiple process, in which migration and invasion are two important processes of it. Tumor cells migration refers to the process that cells migrate from the original position of tumor to a new location under the stimulation of external signals and tumor cells invasion is the process that tumor cells penetrate from their source into the surrounding tissues [5].

Migration and invasion are usually regulated by matrix metalloproteinases (MMPs), which are divided into matrilysins, gelatinase, stromelysins, membrane type MMPs and other MMPs [6]. MMP-9 and MMP-2 belong to gelatinase, and they are the pivotal hydrolytic enzymes involved in hydrolyzing type IV collagen, a central constituent of the ECM and promoted tumor metastasis [1]. In addition, studies have shown that gelatinases can release cryptic information from ECM by degrading ECM, leading to cells migration [7]. Therefore, restraining the MMP-9 and MMP-2 expression is an effective strategy for tumor metastasis treatment.

Generally, the expression of MMP can be regulated in different ways and activating transcription factors can regulate MMP-9 expression [8]. The promoters of MMP-9 have multiple transcription factor-binding motifs, including NF-κB consensus sites [9]. NF-κB, a transcription factor, binds to the κB sequence in DNA, and its activation can increase MMP-9 expression [10]. Furthermore, it has been proved that active MAPK transfers to the nucleus and then activates transcription factors that can interact with transcription factor binding sites in MMP-9 promoters [8]. PI3K/AKT/mTOR signal pathway has also been considered to affect MMP-9 expression [11,12].

Algae is the most important part of marine biological resources, and red algae is the largest group of marine macroalgae in China [13,14]. *Gelidium crinale* is a kind of edible red algae that can not only be eaten raw in salads, soups, meals and condiments, but also produced agar for the food industry. It is rich in polysaccharides, minerals, vitamins and a variety of bioactive compounds and shows high nutritional value. Sulfated polysaccharides, isolated from algae, is a kind of complex polysaccharide containing sulfuric acid group and has many important biological activities. In particular, antitumor activity has attracted a lot of attention. It is of great significance in the industrial fields of biopharmaceutical, food and nutritional health products. However, the chemical properties and biological activities of sulfated polysaccharides may vary according to their composition, molecular size and sulfate content. Different sources of sulfated polysaccharides show different biological activities. Therefore, it is necessary to screen polysaccharides with better performance for application. GNP is the sulfated polysaccharides from *Gelidium crinale* and the molecular of it is 25.8 kD, and the content of sulfate is 16.5% [15]. It is reported that the site and quantity of the sulfate group are closely related to the activity of polysaccharides because sulfate group can promote the activity of it [16]. According to early experiments, GNP has higher sulfate content then most red algae polysaccharides [15]. Therefore, GNP is likely to have high antitumor activity.

In this study, HT1080 cells lines stimulated by phorbol-12-myristate-13-acetate (PMA) were used to be a tumor model. HT1080 cells line with high metastatic has been well studied and widely used in the study of tumor cell metastasis. Furthermore, PMA is an effective tumor promoter that can promote tumor progression related processes. We attempted to assess the inhibitory influence of GNP on the metastasis of HT1080 cells and to assess the influence of GNP on the expression of MMP-9 and MMP-2 and its possible mechanism of MMP-9 regulation in HT1080 cells.

## 2. Materials and Methods

### 2.1. Materials and Chemicals

GNP was extracted from *Gelidium crinale* of Naozhou Island Sea, Zhanjiang City. *Gelidium crinale* was treated with 90% ethanol (*w*/*v* = 1:8), then dried at 45 °C and extracted with 0.1 M HCl (*W*/*V* = 1:8) for 8 h. After neutralizion the extract, centrifugation was performed and the supernatant was concentrated in a rotary evaporator, followed by precipitation with 80% ethanol. The precipitate was redissolved with distilled water and the protein in the solution was remove by Sevag reagent. After concentration and dialysis, the solution was passed through Sepharose Cl-6B column (2.5 × 60 cm), and the elution phase was 0.1 mol/L NaCl. Finally, the collected fractions were freeze-dried to obtain GNP. The purity reached 98.5%. The molecular weight is 25.8 kDa, and galactose is the highest content of its monosaccharide composition. It contains 16.5% of sulfate [15].

HT1080, human fibrosarcoma cell line was purchased from the Cell Bank of the Chinese Academy of Sciences (Shanghai, China). Fetal bovineserum (FBS), trypsin-EDTA (0.25%), dulbecco’s modified eagle’s medium (DMEM), and penicillin/strepto-mycin were from Gibco (New York, NY, USA). PMA was from Sigma-Aldrich (St. Louis, MO, USA). Cell counting kit-8 (CCK-8) was obtained from Zeta Life (San Francisco, SFO, USA). Matrigel was obtained from BD Biosciences (San Jose, CA, USA). p38, p-JNK, p-Ras, Ras, MEK, ERK, p-MEK, p-IκB-α, JNK, p-p38, p-p65, p65, IκB-α, p-ERK, N-cadherin and MMP-9, MMP-2, p-PI3K, β-actin, mTOR, p-AKT, PI3K, AKT, p-mTOR, secondary antibodies were obtained from Santa Cruz Biotechnology Inc. (Dallas, TX, USA) and Cell Signaling Technology (Danvers, MA, USA). 4′,6-diamidino-2-phenylindole (DAPI) was from Shanghai Aladdin Bio-Chem Technology Co., Ltd. (Shanghai, China).

### 2.2. Cells Activity Assay (CCK-8)

HT1080 cells were cultured in different concentrations of GNP (0, 10, 50, and 100 µg/mL) for 48 h after seeding in 96-well plate for 24 h. Then removed the supernatant and added 100 µL DMEM containing 10 µL CCK-8 working fluid to the well and cultured for 1 h in dark and 37 °C. The absorbance was determined at 450 nm by a microplate reader (BioTek, Winooski, VT, USA).

### 2.3. Cell Wound Healing Assay

Applying a sterile tip to make a wound on the 24-well plate that was full of HT1080 cells, followed by cells were treated with GNP and induced with PMA after 1 h. A total of five groups were set up in the experiment. The blank group did not do any treatment, the control group only treated with PMA (10 ng/mL), and the sample group added PMA (10 ng/mL) and GNP of different concentrations (10, 50 and 100 µg/mL). The following experiments are in the same group settings. Migration was recorded at 0 h, 12 h and 24 h, respectively, using a microscope (Olympus, Tokyo, Japan).

### 2.4. Trans Well Matrigel Invasion Assay

Placed the 24-well place (NEST biotechnology, Wuxi, China) at 37 °C for 30 min after adding Matrigel to the upper chamber of it. Then 200 µL cell suspension containing GNP (0, 10, 50, and 100 µg/mL) was added to the chamber above and stimulated with PMA. Furthermore, 500 µL complete medium was put into the chamber below. Cell suspension and Matrigel were removed after 24 h and the cells were stained with DAPI and recorded by a fluorescence microscope.

### 2.5. Gelatin Zymography

Cells was cultured with GNP for 1 h, then treated with PMA. The upper cells culture medium was collected after 48 h. An equal volume of cells medium of different experimental groups was separated with 10% separating gel that contained 1% gelatin. The gel was washed 3 times in the eluent (50 mM Tris, 2.5% Triton X-100, 5 mM CaCl_2_) at the end of electrophoresis. Then, it was plated in the incubation solution (200 mM NaCl, 0.02% Brij-35, 5 mM CaCl_2_, 50 mM Tris,) to incubate for 60 h at 37 °C. Next, the gel was stained by used of Coomassie Brilliant Blue and the results were recorded by the gel imaging system.

### 2.6. Quantitative Real Time PCR (qPCR)

Using RNA-easy^TM^ Isolation Reagent (Vazyme, Nanjing, China) to extract the total RNA and the purified RNA was converted to cDNA by the used of HiScript II 1st Strand cDNA Synthesis Kit (+gDNA wiper) (Vazyme, Nanjing, China). qPCR was performed by CFX96 Real-Time System (BIO-RAD, Hercules, CA, USA). The primers used were shown in Table 1.

### 2.7. Immunocytochemistry

Cells were pretreatment with GNP for 1 h and then treated with PMA for 24 h. Using 4% paraformaldehyde to fix cells for 30 min and PBS containing 0.2% Triton X-100 to permeabilize cells for 15 min, then cells were incubated with 5% BSA for 1 h and the primary antibody at 4 °C overnight. Subsequently, cells were incubated with secondary antibodies in dark and stained with DAPI. Finally, the results were recorded by an inverted fluorescence microscope

### 2.8. Western Blot

Proteins are obtained by lysing cells with radio immunprecipitation assay lysis buffer containing 1% phenylmethylsulfonyl fluoride. Then the protein is quantified using a BCA protein assay kit, separated using SDS-PAGE, and shifted to NC membrane. The membrane was incubated with milk, primary antibody and secondary antibody in order. Finally, using an enhanced chemiluminescence detection system (Syngene, Cambridge, UK) to evaluate the level of protein expression.

### 2.9. Statistical Analysis

All values are expressed as the mean ± standard deviation (*n* = 3), and statistical analyses between different groups were performed by one-way ANOVA. The statistical significance of all trials was set top < 0.05.

## 3. Results

### 3.1. Effect of GNP on the Viability of HT1080 Cells

**Figure 1:**
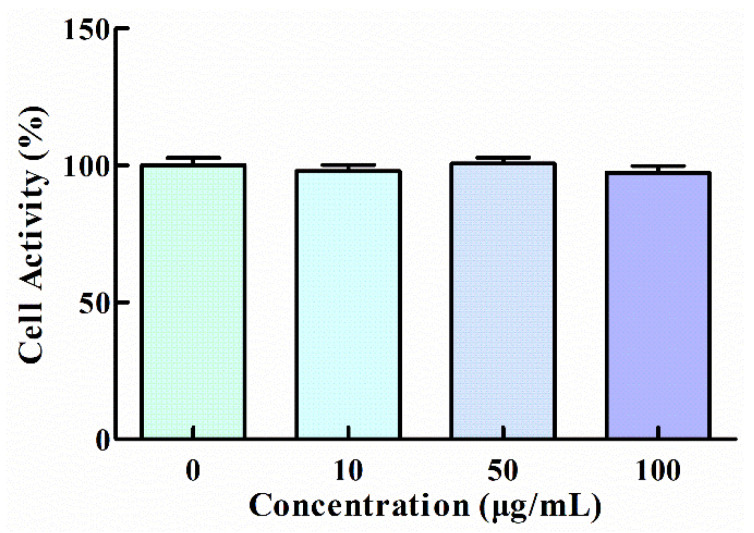
Effect of GNP on the viability of HT1080 cells. Data are shown as mean ± SD (*n* = 3).

The cytotoxicity of GNP to HT1080 cells was tested by CCK-8 assay and the result showed that any significant difference was not observed in cell viability between GNP treated and untreated cells, which indicates that GNP with a concentration of up to 100 μg/mL has no toxic effect (Figure 1). Thus, the concentrations (10, 50, and 100 μg/mL) of GNP were selected for the experiments subsequently.

### 3.2. Effect of GNP on the Migration and Invasion Ability of HT1080 Cells

**Figure 2:**
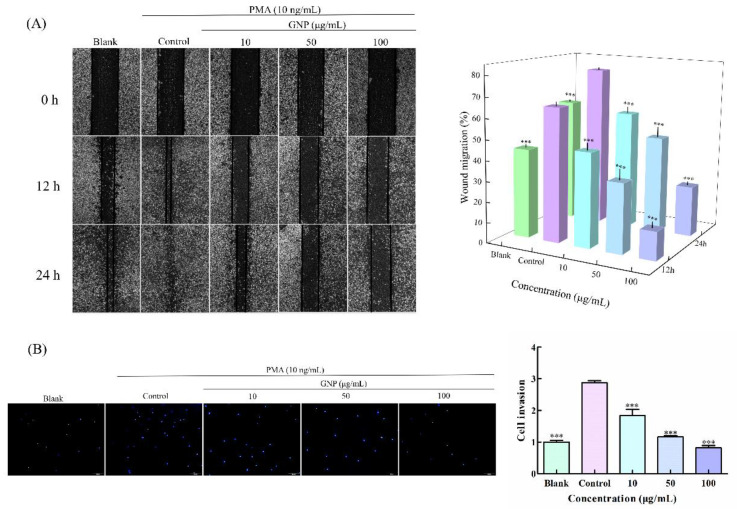
(**A**) The influence of GNP on HT1080 migration. Scraping the confluent cell monolayer to create the wounds, and the migration was observed at 0, 12 and 24 h, respectively. (**B**) The effect of GNP on HT1080 cells invasion. Data are shown as mean ± SD (*n* = 3), *p* < 0.001 (***) compared with PMA alone treated control group.

To detect the impact of GNP on HT1080 cells metastasis, wound healing assay (Figure 2A) and trans well cells invasion assay (Figure 2B) was carried out. In wound healing assay, the cells of the control group migrated to the position of wound rapidly, while after treating with GNP (10, 50 and 100 μg/mL), the wound became difficult to close. The inhibition rates of HT1080 cells migration were 29.3 ± 7.3%, 48.2 ± 7.5% and 78.7 ± 5.2%, respectively, when cells were treated with GNP at 10, 50 and 100 μg/mL at 12 h compared with the control group and at 24 h the inhibition rates were 24.4 ± 4.6%, 48.0 ± 6.8% and 66.4 ± 2.1%, respectively. The dates indicated that GNP could effectively inhibit the cells migration, and the inhibition effect increases with the increase of concentration. In the trans well cells invasion assay, the number of cells induced by PMA only penetrating the matrix gel was the largest, while it reduced greatly in the group treated with GNP. After 24 h treatment with GNP, the invaded cells were decreased by 36.3 ± 10.3%, 59.7 ± 0.9% and 71.4 ± 5.4% at 10, 50 and 100 μg/mL, respectively, compared with the control group. The results indicated that GNP can significantly suppress the invasion of HT1080 cells. Migration and invasion are key steps in metastasis, and substances that inhibit migration and invasion may prove to have better antitumor effects, as metastasis is often present when malignant tumors are found in humans. Therefore, GNPs that can inhibit migration and invasion of HT1080 cells may have great potential in antitumor and it is necessary to further study.

### 3.3. Effect of GNP on the Expression and Activity of MMP-9 and MMP-2 in HT1080 Cells Induced by PMA

**Figure 3:**
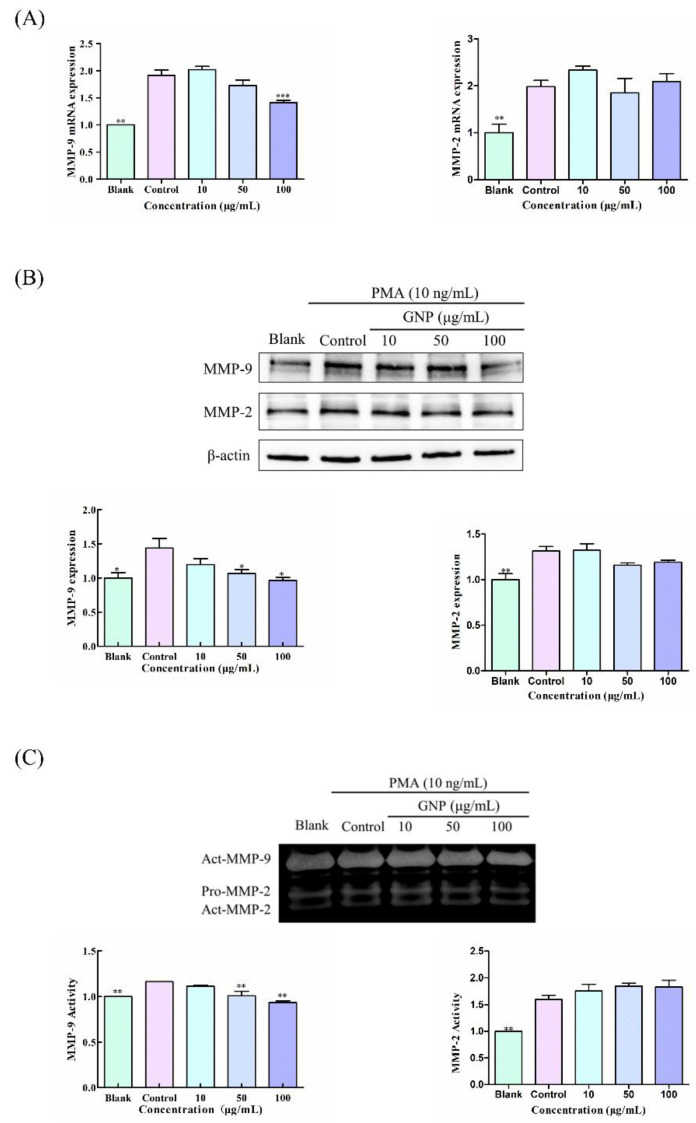
(**A**) The MMP-9 and MMP-2 mRNA levels in HT1080 cells. β-actin was the loading control. (**B**) The protein of MMP-9 and MMP-2 level in HT1080 cells. β-actin was the loading control. (**C**) The enzymatic activity of MMP-9 and MMP-2 in HT1080 cells supernatant. Data are shown as mean ± SD (*n* = 3). *p* < 0.05 (*), *p* < 0.01 (**), *p* < 0.001 (***) compared with PMA alone treated control group.

The influence of GNP on MMP-9 and MMP-2 expression and activity in HT1080 cells induced by PMA was studied by qPCR, western blot assay and gelatin zymography assay. In qPCR and western blot assay, GNP can obviously decrease the mRNA and protein of MMP-9. The MMP-9 mRNA and protein level of HT1080 cells treated with PMA increased by 91.1 ± 14.5% and 43.7 ± 9.0%, respectively, compared to the blank group, whereas treatment with GNP at doses of 100 μg/mL, the mRNA and protein of MMP-9 decreased by 25.5 ± 7.0% and 31.2 ± 8.9%, respectively, compared to the control group (Figure 3A,B). In addition, in zymography analysis (Figure 3C), the control group stimulated by PMA secreted more MMP-9 to hydrolyze the surrounding gelatin, while after adding GNP, the secretion of MMP-9 decreased, resulting in the reduction of the hydrolysis of the gelatin. MMP-9 activity dropped by 19.5 ± 2.0% when HT1080 cells were treated with 100 μg/mL of GNP compared to the control group. On the other side, GNP had no marked influence on the MMP-2 expression and activity. MMP-9 is a pivotal enzyme that involved in ECM degradation, and its overexpression is closely related to tumor metastasis. In addition, GNP can inhibit the expression of MMP-9, suggesting that GNP may have anti-tumor activity by regulating MMP-9.

### 3.4. Effect of GNP on the Expression of N-Cadherin in HT1080 Cells by PMA

**Figure 4:**
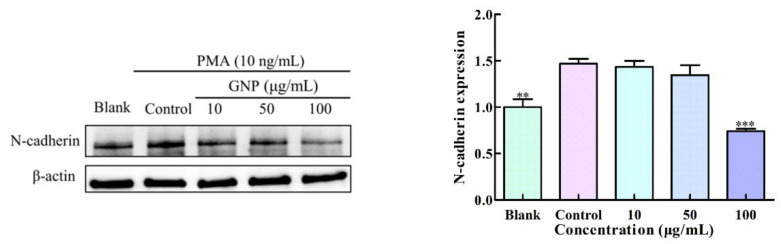
The expression of N-cadherin in HT1080 cells. β-actin was the loading control. HT1080 cells were treated with different concentrations of GNP. Data are shown as mean ± SD (*n* = 3). *p* < 0.05 (*), *p* < 0.01 (**), *p* < 0.001 (***) compared with PMA alone treated control group.

N-cadherin is a calcium-binding, single-pass transmembrane glycoprotein correlated with metastasis, and it can produce signals leading to the up regulation of MMP-9. In this research, the protein level of N-cadherin was measured through western blot assay (Figure 4), The result shows that PMA can activate the overexpression of N-cadherin, while GNP can effectively down its the expression. GNP (100 μg/mL) treatment resulted in 49.5 ± 2.5% decreases in N-cadherin protein level of HT1080 cells compared to the control. The dates indicates that GNP is able to regulate the N-cadherin.

### 3.5. Effect of GNP on NF-κB Pathway in PMA-Induced HT1080 Cells

**Figure 5:**
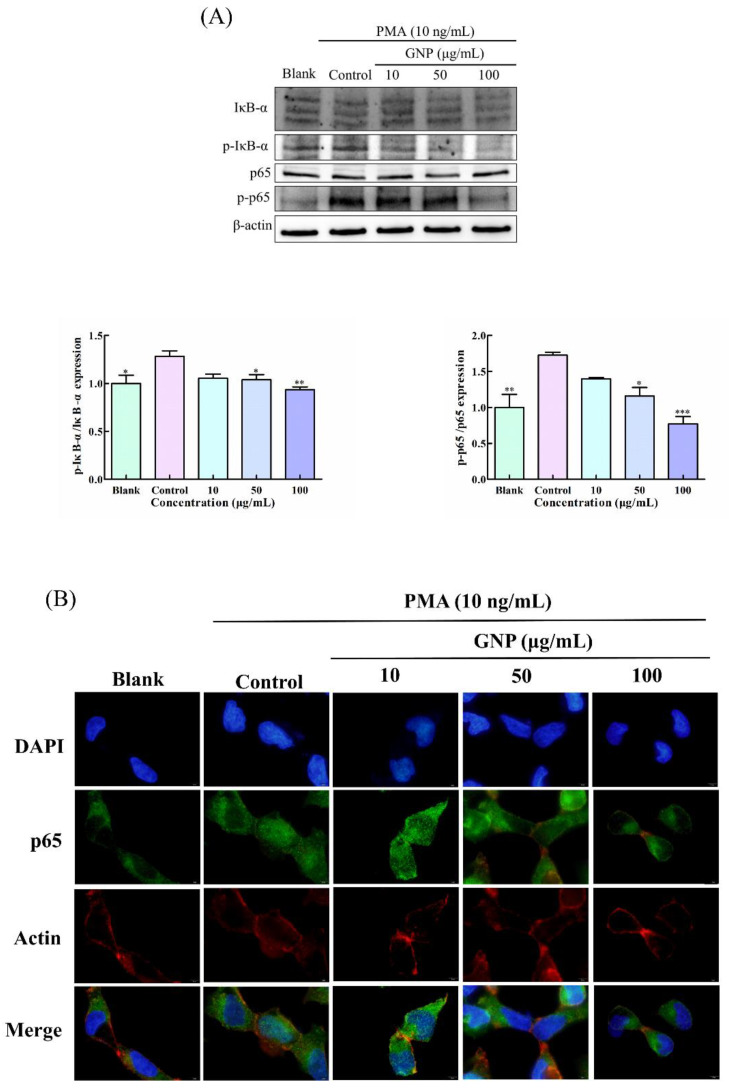
(**A**) The protein expression level of NF-κB signaling pathway in HT1080 cells. β-actin was used as an internal control. (**B**) Nuclear translocation of p65 was observed through an overlay of green p65 staining with blue DAPI staining and red cytoskeleton staining. Data are shown as mean ± SD (*n* = 3). *p* < 0.05 (*), *p* < 0.01 (**), *p* < 0.001 (***) compared with PMA alone treated control group.

NF-κB is considered as a significant transcription factor that can regulate the expression of MMP-9 and has been found to relate to cells migration and invasion [17]. Western blot analysis (Figure 5A) and immunocytochemistry (Figure 5B) were conducted to assess the impact of GNP on NF-κB pathways in HT1080. The results show that PMA can elevate the phosphorylation of p65 and IκB-α. However, the phosphorylation activation of p65 and IκB-α was obviously suppressed after treating with GNP. Compared to the control group, the phosphorylation level of p65 and IκB-α were dropped by 55 ± 6.7% and 26.5 ± 7.8%, respectively, when treated with GNP at 100 μg/mL. Moreover, the results of p65 translocation analysis, GNP can effectively prevent p65 nuclear translocation. The result shows that GNP can effectively suppress NF-κB signaling.

### 3.6. Effect of GNP on MAPK Pathway in PMA-Induced HT1080 Cells

**Figure 6:**
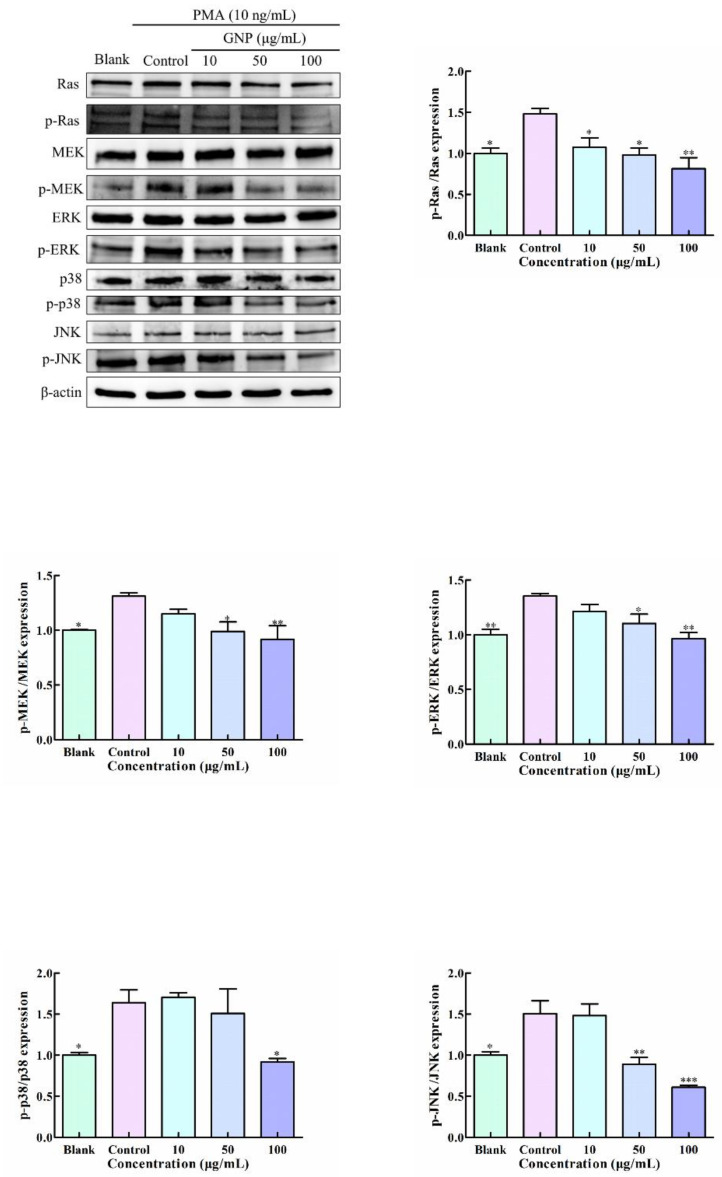
The protein level of MAPK signaling pathway in HT1080 cells was determined by western blot analysis. β-actin was used as an internal control. Data are shown as mean ± SD (*n* = 3). *p* < 0.05 (*), *p* < 0.01 (**), *p* < 0.001 (***) compared with PMA alone treated control group.

Abnormal MAPK signaling pathway partakes in the progression of many malignancies. There are four types of MAPK in eukaryotic cells: ERK, p38, JNK and ERK5. Among them, Ras/Raf/MEK/ERK signaling pathway plays a significant part in all MAPK signal pathways [18]. Studies have proved that the activation of ERK, p38 and JNK pathways contribute to the upregulation of MMP-9 [12,19]. To determine whether GNP regulates the expression of MMP-9 through MAPK pathway, the phosphorylation levels of core proteins of three branches: Ras/Raf/MEK/ERK, JNK, and p38 involved in MAPK pathway were tested after treatment with GNP of the cells. It shows that (Figure 6) the protein level of p-RAS, p-MEK, p-ERK, p-JNK and p-p38 in HT1080 cells were decreased after treatment with GNP. Compared with the PMA-induced group, the phosphorylation levels of ERK, JNK RAS, and p38 decreased to 44.5 ± 14.1%, 28.8 ± 4.1%, 58.7 ± 6.9% and 43.2 ± 6.9% when treated with GNP at 100 μg/mL. The result illustrated that GNP could inhibit the phosphorylation of Ras/Raf/MEK/ERK, JNK, and p38 MAPK pathway.

### 3.7. Effect of GNP on PI3K/AKT/mTOR Pathway in PMA-Induced HT1080 Cells

**Figure 7:**
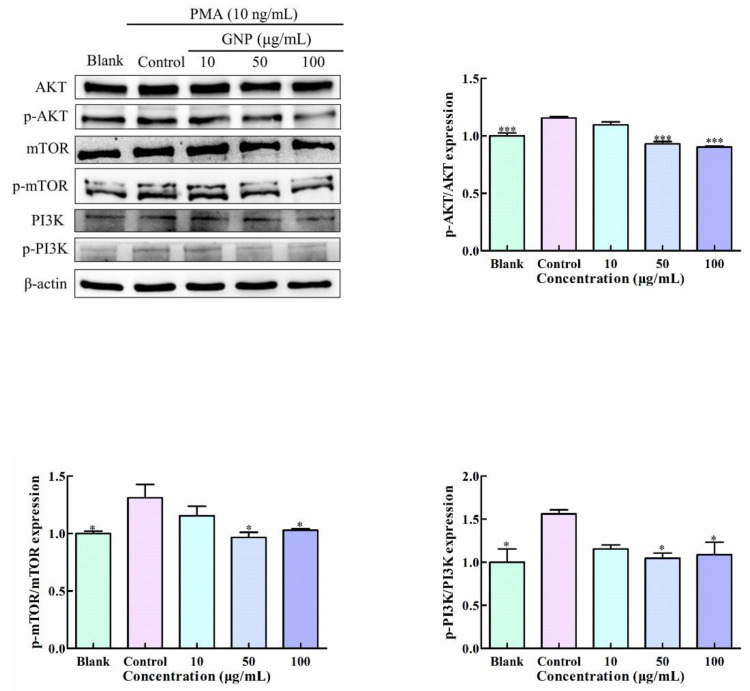
The protein level of PI3K/AKT/mTOR signaling pathway in HT1080 cells was determined by western blot analysis. β-actin was used as an internal control. Data are shown as mean ± SD (*n* = 3). *p* < 0.05 (*), *p* < 0.001 (***) compared with PMA alone treated control group.

PI3K/AKT/mTOR pathway consists of three main driving molecules: PI3K, AKT and rapamycin mammalian target (mTOR). The western blot assay was implemented to determine the impact of GNP on the PI3K/AKT/mTOR signaling activities in HT1080 cells. In Figure 7, PMA-induced significantly elevated phosphorylation of p-AKT, p-PI3K, and p-mTOR. However, GNP treatment can effectively suppress phosphorylation activation. GNP with the concentration of 100 μg/mL showed 30.3 ± 13.3%, 21.9 ± 0.02%, and 20.1 ± 11.1% inhibition of the phosphorylation of PI3K, AKT and mTOR, respectively. These results showed that the treatment of GNP can effectively block PI3K/AKT/mTOR pathway in HT1080 cells.

## 4. Discussion

Tumor is a main cause of human death, and it is tumor metastasis that makes it difficult to cure. Therefore, it is extremely important to explore effective drugs for blocking metastasis. Red algae contain many important bioactive compounds, among which sulfated polysaccharides is one. It had proved to possess a sea of efficacy, especially in anti-tumor, and it is one of the most common polysaccharides in cancer treatment [20].

*Gelidium crinale* (Naozhou Island Sea, Zhanjiang City) belongs to red algae, and GNP is sulfated polysaccharide extracted from it. Previous studies have shown that compared with other red algae polysaccharides, GNP contains more sulfate groups, which is the main functional and active group. Therefore, we speculated that GNP has stronger anti-tumor activities. In this experiment, we selected the concentrations (10, 50 and 100 µg/mL) in which GNP had not toxic to HT1080 cells. The results show that GNP markedly inhibited the metastasis of HT1080 cells, and the inhibitory effect increased with the doses increasing. When the concentration of GNP was as low as 10 μg/mL, GNP showed an evidently inhibitory effect on the migration and invasion of HT1080 cells. Compared with other sources of SP in inhibiting migration and invasion (Table 2), GNP has no less or better anti-tumor metastasis activity than others. Therefore, it may have great development potential.

MMPs, especially MMP-9 and MMP-2, can digest the ECM and facilitate the metastasis of tumor cells. Therefore, MMPs can be used as therapeutic targets to exploit drugs against tumor metastasis. Jun at al. [21] showed that quercetin can inhibit the expression of MMP-2, MMP-9 and p-Akt1, in HCCLM3 cells, thereby weakening cells migration and invasion. Lee et al. [22] showed that dihydroavenanthramide D inhibited cancer cell invasion by decreasing MMP-9 expression. Although many studies have studied MMP inhibitors, there are no MMP inhibitors that have really been used in clinical treatment and achieved good therapeutic effects in cancer metastasis, and one reason for this is the side effects of MMPs inhibitors. Therefore, it still needs to further explore safer and more effective MMP inhibitors. In this study, GNP suppressed the MMP-9 mRNA expression at a transcriptional level and inhibited the MMP-9 protein level and enzyme activity in HT1080 cells but had no significant impact on MMP-2. Therefore, GNP is likely to become an MMP-9 inhibitor that can effectively treat tumor metastasis.

Subsequently, we investigated the underlying mechanism of GNP regulation of MMP-9 in HT1080 cells. Regulation of MMP-9 closely connects with transcription and translation [23]. NF-κB, which can directly regulate the transcription of MMP-9, is most often retains in the cytoplasm in an inactive form by binding IκB. When IκB is phosphorylated, the proteasomal of IκB will be degraded, and then NF-κB is allowed to translocate to the nucleus and completes the transcription of the target genes [24,25]. The results show that GNP can inhibit the phosphorylation of NF-κB in HT1080 cells induced by PMA, and it can decrease the nuclear translocation of the p65 subunit. This means that GNP can inhibit the expression of MMP-9 by inhibiting NF-κB pathway.

In addition, activating NF-κB can heighten the transcription of N-cadherin. N-cadherin is a pivotal marker of epithelial-to-mesenchymal transition (EMT) associated with tumor cells survival, migration and invasion [26]. Da et al. [27] assessed the relationship between N-cadherin expression and MMP-9 expression, the results show that N-cadherin knockdown could reduce the transcription of MMP-9. N-cadherin would enhance the expression of MMP-9, and then promote the malignant progression and invasion [26]. From the study, we found that GNP is able to decrease the expression of N-cadherin in HT1080 cells induced by PMA, this shows that GNP may inhibit the MMP-9 through reducing the expression of N-cadherin.

MAPK is an upstream modulator of NF-κB that can adjust a series of cellular reaction, such as cell growth, survival and death. In Ras/Raf/MEK/ERK pathway, Ras is an upstream protein, and its activation can initiate the phosphorylation of Raf [28]. Raf shows activity after binding to Ras, and continues to activate downstream and then eventually leads to ERK over activated. In this experiment, the phosphorylation levels of Ras, MEK and ERK increased when HT1080 cells were induced by PMA, but were significantly inhibited after treatment with GNP. This indicates that GNP can inhibit the activation of Ras/Raf/MEK/ERK pathway. Furthermore, GNP also inhibited the phosphorylation of p38 and JNK. It is reported that the ERK, JNK and p38 MAPK can affect MMP-9 via modulating NF-κB activity, and the expression and activity of MMP-9 can be reduced through blocking the activation of MAPKs signaling pathway [29,30]. These results suggest that GNP can inhibit MAPK pathway and thus inhibit the MMP-9.

Similarly, PI3K/AKT/mTOR has been proved to be involved in metastasis in malignancies. The mechanism of PI3K/AKT/mTOR activation leading to increased proliferation, invasion and migration seem to be through the regulation of MMPs [31]. Hua et al. [11] indicate that ruscogenin can reduce the MMP-9 expressions by regulating PI3K/AKT/mTOR pathway, thereby blocking the metastasis of hepatocellular carcinoma. This study discussed the influence of GNP on the activated PI3K/AKT/mTOR pathway. Our data exhibited that GNP inhibits the phosphorylation of PI3K/AKT/mTOR pathway, and the impact of GNP on the expression of MMP-9 is considered to be closely related to this signaling pathway.

In summary, GNP can inhibit the MMP-9 via NF-κB, MAPK and PI3K/AKT/mTOR pathways. It is well known that MMP-9 has a close link with metastasis, and inhibiting the expression of MMP-9 can prevent tumor metastasis. Our studies show that the effect of GNP on tumor cells metastasis may closely relate to the regulation of GNP on MMP-9.

In addition, sulfated polysaccharide is a polyanion linear macromolecular compound containing sulfuric acid group, which shows a wide range of biological activities [32]. Among them, antitumor is one of the attractive biological activities of sulfated polysaccharides, and the activity intensity is affected by the monosaccharide composition and number, the type of glycosidic bongs, molecular weight and the number and position of sulfate group and so on [33].

The antitumor activities of polysaccharides with different sugar units vary greatly. It is reported that some heteropolysaccharides primarily consist of galactose (Gal), rhamnose (Rha), mannose (Man), xylose (Xyl) arabinose, (Ara) and other monosaccharides have anticancer activity, and our early studies have shown that GNP is a mixture of different polysaccharides mainly containing Gal (65.05%), Xyl (11.55%), glucose (6.73%), ribose (0.47%), fucose (11.19%), arabinose (0.26%), glucuronic acid (5.54%), amino galactose (0,43%), and Rha (0.79%) [15,34]. This may be the basis of GNP antitumor activity. Furthermore, the configuration of glycosidic bonds also affects sugar activity and generally, the polysaccharides with β-configuration has better activity. Fourier transform infrared spectroscopy indicates the presence of βconfiguration in GNP, and this may also be one of the reasons why GNP has better activity. In addition, the molecular weight of polysaccharide is also considered as a significant factor affecting polysaccharide activity [35]. Generally, polysaccharides with a molecular weight between 20–500 kDa have significant anticancer activity, while too small or too large of molecular weight will limit its activity [34]. GNP’s molecular weight is 25.8 kDa, which is just in this range. Sulfate groups of SP play a critical role in its biological activity, which can interact with various cationic proteins. It is usually more bioactive due to the existence of sulfate groups [36]. GNP contains 16.50% sulfate, while the sulfate content of red algae polysaccharide is about 10% [15]. Therefore, the excellent antitumor activity of GNP may be related to sulfate groups.

In this study, we explored the antitumor effect of GNP. GNP has obvious inhibitory effect on tumor cell migration and invasion, which are the important steps in metastasis. In addition, GNP can inhibit the expression and activity of MMP-9, which can mediate tumor metastasis. Metastasis is main feature of malignant tumors. Therefore, we speculate that GNP has better antitumor potential. However, the more specific relationship between GNP structure and anti-tumor metastasis activity is unclear, and it is not clear whether GNP inhibits other MMP at the same time and has an impact on other physiological processes, and whether GNP has the same significant anti-tumor effect in vivo trials. These still need to be further studied.

**Table 2:** Effects of sulfated polysaccharides from different sources on migration and invasion.

Source	Cell	Effective Concentration of Migration or Invasion	Mechanism	Reference
*Antrodia cinnamomea*	Human lung cancer A549 cells	Migration: 200 μg/mL	Promoting degradation of TGFRs and inhibiting Smad and non-Smad signaling pathways.	[37]
*Gracilaria fisheri*	CCA cells (HuCCA-1 and RMCCA-1) established from CCA tissue fragments of Thai patients	Migration: 10 μg/mL	Mediated by inhibition of MAPK/ERK signal transduction pathway	[38]
*Codium isthmocladum*	B16-F10 murine melanoma cell line	Invasion: 100 μg/mL	-	[33]
*Undaria pinnatifida*	OC cell lines (SKOV3, A2780)	Migration and Invasion: 100 μg/mL	Inhibits Hh pathway conduction in OC cells	[39]
*Undaria pinnatifida sporophylls*	Mouse Hca-F hepatocarcinoma cell line	Invasion: 500 μg/mL	Mediated through the mechanism involving inactivation of the NF-κB pathway	[40]
*Stichopus variegatus*	Human breast cancer cells MDA-MB-231	Migration: 100 ug/mL	-	[41]
*Ascophyllum nodosum*	Murine B16 melanoma	Migration: 10 ug/mL Invasion: 5 ug/mL	Inhibition of EMT, reduced the expression of MMP-9	[42]

## 5. Conclusions

This research revealed the inhibitory impact of GNP on the metastasis of HT1080 cells and the inhibition of MMP-9 overexpression for the first time. The results indicated that GNP prevents tumor metastasis effectively and is an effective inhibitor of MMP-9. GNP inhibits MMP-9 by adjusting NF-κB, MAPK and PI3K/Akt/mTOR signaling pathways and reducing the expression of N-cadherin. MMP-9 plays an important role in the process of tumor development, especially in tumor metastasis. Exploring its inhibitors is one of the important directions in the research of antitumor drugs. At present, many MMP inhibitors have been designed for anti-tumor, but the therapeutic effect of these compounds is limited, and most clinical data are disappointing. It is necessary to find new and effective MMP inhibitors. As a newly discovered MMP-9 inhibitor, GNP has excellent inhibitory effect on migration and invasion, and may have great potential in future anti-tumor therapy. This study provides a theoretical and scientific basis for the application of GNP in tumor metastasis and MMP-9 expression.

## Figures and Tables

**Table 1:** Details of primers of qPCR detection.

Primer	Primer Sequence (5′-3′)
MMP-9	F: 5′-TCCTGGTGCTCCTGGTGCTG-3′
	R: 5′-CTGCCTGTCGGTGAGATTGGTTC-3′
MMP-2	F: 5′-AGCCAAGCGGTCTAAGTCCAGAG-3′
	R: 5′-GGAATGAAGCACAGCAGGTCTCAG-3′
β-actin	F: 5′-CCTGGCACCCAGCACAAT-3′
	R: 5′-GGGCCGGACTCGTCATAC-3′

## Data Availability

The data presented in this study are available on request from the corresponding author.
